# Hydatid cysts of the pelvis following pulmonary hydatid disease: A rare case report

**DOI:** 10.1016/j.heliyon.2021.e08056

**Published:** 2021-09-22

**Authors:** Jafar Ganjipour Sales, Hamed Behniafar, Mohammadreza Bazavar, Mojtaba Varshochi, Abbas Majdi Seghinsara

**Affiliations:** a Department of Orthopedics Surgery, Shohada Hospital, Tabriz University of Medical Sciences, Tabriz, East Azerbaijan, Iran; b Department of Medical Parasitology, Sarab Faculty of Medical Sciences, Sarab, East Azerbaijan, Iran; c Department of Orthopedic Surgery, Faculty of Medicine, Tabriz University of Medical Sciences, Tabriz, East Azerbaijan, Iran; d Tropical and Infectious Diseases Research Center, Tabriz University of Medical Sciences, Tabriz, East Azerbaijan, Iran; e Department of Anatomical Sciences, Faculty of Medicine, Tabriz University of Medical Science, Tabriz, East Azerbaijan, Iran

**Keywords:** *Echinococcus granulosus*, Osseous hydatid cyst, Pelvis, Surgery

## Abstract

**Background:**

Echinococcosis is a zoonotic parasitic infection, caused by the larval stage of *Echinococcus* species, especially *Echinococcus granulosus*. This parasite can develop cysts in different organs of the human body.

**Case report:**

An osseous hydatid cyst is an uncommon and rare phenomenon. Here, we report a rare case of an osseous cyst in a 49-year-old woman with pain in the sacral region and a history of hydatidosis. Computed tomography (CT) and magnetic resonance imaging (MRI) were performed for the patient. The chest CT scan and MRI results indicated masses in the pelvis. According to the patient's clinical signs, CT and MRI findings, and clinical history, osseous hydatidosis was established as the final diagnosis. Accordingly, surgical removal of cysts and chemotherapy were applied for treatment. The removed cysts were also sent for pathological examinations, which confirmed the diagnosis.

**Conclusion:**

Surgical removal is essential for the treatment of hydatidosis, and adjuvant chemotherapy is crucial for a better prognosis.

## 1 Introduction

Hydatidosis is a zoonotic parasitic infection, caused by the larval stage of *Echinococcus granulosus* (*E. granulosus*), a canine taeniid tapeworm. Among species belonging to the genus *Echinococcus*, two species, namely, *E. granulosus*, as the causative agent of cystic echinococcosis (CE), and *E. multilocularis*, as the causative agent of alveolar echinococcosis, are of greater importance clinically [1].

The disease has a worldwide distribution. In the life-cycle of these heteroxenous parasites, dogs (and other canines) and ruminates are definitive and intermediate hosts, respectively. Besides, *E. granulosus* can infect humans as accidental intermediate hosts. The infection is transmitted to humans by helminth eggs through close contact with infected dogs or contaminated fresh food products. After egg hatching, the larvae penetrate into the intestines and distribute in various organs through the blood or lymphatic system [1]; eventually, cysts are formed.

Although CE may develop in different organs, it mostly affects the liver and the lungs, respectively. In addition to mentioned organs, other can be affected, for example retroperitoneal cyst such as pancreatic cysts, and parietal cysts were reported [2, 3, 4]. Osseous CE is extremely rare, accounting for about 0.5%–2.5% of all human cases [5, 6]. The most common sites of osseous cysts are the femur and the tibia, respectively [6, 7]. The pelvis is another frequently affected area, following the long bones. Evidence shows that pelvic cysts account for 16%–28% of all cases of osseous hydatidosis [6].

## 2 Case report

In February 2021, a 49-year-old woman was admitted to Shohada Hospital in Tabriz, Iran, with a complaint of pain in the sacral region. The patient had a history of surgery due to pulmonary hydatidosis and osseous hydatidosis in the left hip four years ago. She was from a rural area in Hashtrood County, East Azerbaijan Province, Iran, and had close contact with dogs and sheep. She had experienced progressive swelling in the sacral region for one year. Written consent was obtained from the patient for using the images and publishing this case report.

## 3 Methods

On admission, the analysis of blood samples revealed a normal range for hematocrit (38.1%), hemoglobin level (12.6 g/dL), erythrocyte sedimentation rate (ESR: 17 mm/h), platelet count (200,000/μL), and WBC count (10,800 WBCs/μL); however, neutrophilia (71.2%) and lymphocytopenia (23.3%) were detected. Also, the C-reactive protein was measured to be 1+.

**Figure 1:**
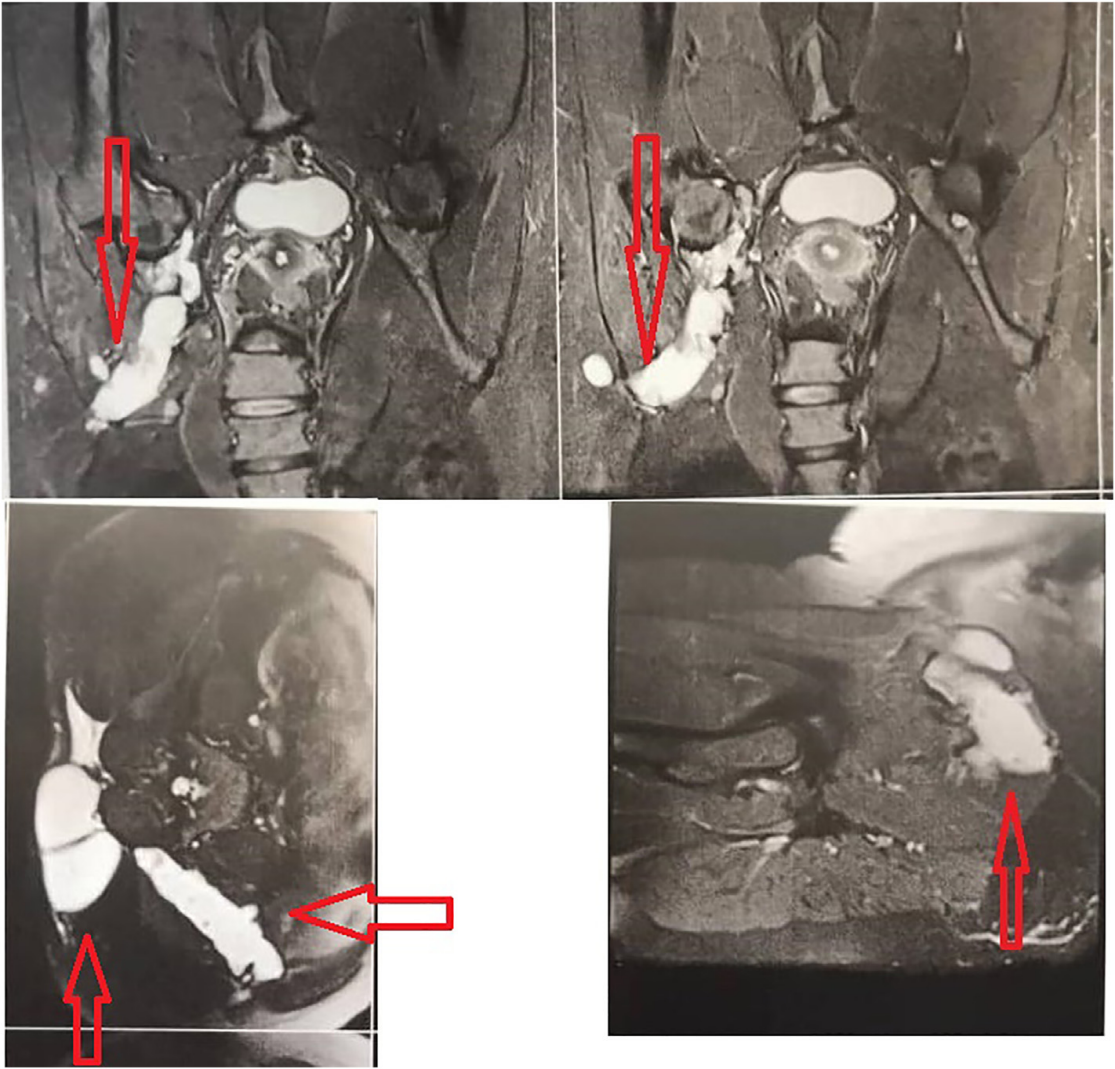
CT scan and MRI of the pelvis showing cysts (red arrows).

Computed tomography (CT) and magnetic resonance imaging (MRI) were carried out for the patient (Figure 1). The CT and MRI results indicated masses in the soft tissue of the sacrum, inside the sacrum (in the wings of S1 and S2 vertebrae), and most parts of the medulla oblongata in the left ilium. The largest cyst size was measured at approximately 10 × 4 cm. The previous chest radiograph, acquired before the last surgery, showed nodular lesions in the lower lobes of both right and left lungs. A diffuse mass was observed in the iliac bone, spreading to the posterior soft tissue, which was suspected to be a hydatid cyst.

According to the patient's clinical signs, CT and MRI findings, and medical history, the final diagnosis of osseous hydatidosis was established. Surgery was the treatment of choice. Radical excision of the soft tissue and curettage of the iliac wing were performed, and the bone was filled with cement. Under the central operating room conditions and general anesthesia, the skin and subcutaneous tissue were cut using a posterior approach. After making an incision, the larger cyst of the soft tissue and cysts of the left wing of the ilium, which also affected the sacroiliac joint, were carefully removed.

After removing the cysts, the area was curetted; a hypertonic solution was injected; the area was rinsed extensively with normal saline and ethanol; and bone defects were filled with cement. Moreover, chemotherapy was initiated with oral albendazole (400 mg twice a day) prior to surgery and continued for four weeks after surgery. The removed cysts were sent for pathological examinations, which also confirmed the diagnosis of hydatidosis. Chemotherapy was continued for one month after surgery.

## 4 Discussion

Hydatidosis is a worldwide zoonotic disease and a global public health problem. Hydatid cysts grow gradually and can be usually found in visceral organs. They are endemic in developing countries, such as Iran, where sheep breeding is common. The highest prevalence of CE has been reported in temperate-zone countries, including Central Asian countries, China, Australia, the Mediterranean region, and parts of South America [8].

As mentioned earlier, osseous hydatidosis is a rare phenomenon. This disease may remain asymptomatic for years, and its clinical symptoms are often insignificant. It results in the replacement of the trabecular bone by various slow-growing vesicles that proliferate along the medullary canal, without creating encapsulated cysts similar to other organs; therefore, diagnosis can be delayed [9]. Osseous hydatidosis is usually diagnosed in advanced stages when the cyst has enlarged and caused complications, such as infection or abscess fistula, neural deficits, osteolytic bone destruction, invasion to soft tissues, and pathological fractures [6, 10].

The differential diagnoses for osseous hydatidosis are broad and include osteosarcoma, chronic osteomyelitis, chondrosarcoma, osteoclastoma, some tumors, such as aneurysmal cysts, metastases, and tuberculosis [11]. Besides, pelvic hydatid cysts can be dangerous, because they remain asymptomatic for some years, allowing them to spread to other parts of the body, such as the hips and sacroiliac joints [12]; this invasion makes it almost impossible to eradicate the parasite.

The surgical removal of osseous cysts is the primary treatment method; however, due to the high recurrence rate, chemotherapy is also recommended along with surgery, and replacing damaged bone with artificial bone [13, 14, 15]. Therefore, we used the surgical method and tried to eradicate the parasite using albendazole. Oral chemotherapy with Albendazole was continued for four weeks to prevent the possibility of cyst regrowth from surgical debris in the surrounding soft tissues [16]. In our case, according to the patient's history, both a new infection (because of close contact with animals) and a secondary infection due to a previous cyst were possible.

## 5 Conclusion

Although osseous hydatidosis is not a malignancy, it is a severe disease that should be differentiated from diseases with a poor prognosis.

## Declarations

### Author contribution statement

All authors listed have significantly contributed to the investigation, development and writing of this article.

### Funding statement

This research did not receive any specific grant from funding agencies in the public, commercial, or not-for-profit sectors.

### Data availability statement

Data included in article/supplementary material/referenced in article.

### Declaration of interests statement

The authors declare no conflict of interest.

### Additional information

No additional information is available for this paper.
